# Mental health in China: exploring the impacts of built environment, work environment, and subjective perception

**DOI:** 10.3389/fpsyg.2024.1352609

**Published:** 2024-02-22

**Authors:** Zhou Fang, Yu Lin, Chuangyuan Chen, Jian Jiang, Letian Dong

**Affiliations:** ^1^Guangzhou Transport Planning Research Institute Co., Ltd., Guangzhou, China; ^2^School of Architecture and Urban Planning, Guangdong University of Technology, Guangzhou, Guangdong, China

**Keywords:** mental health, migrants, built environment, work environment, subjective perception

## Abstract

**Introduction:**

The shifting living and working conditions have profound impacts on the residents’ mental health. However, current research in this field has not remarkable investigated.

**Methods:**

This study used the China Labor-force Dynamic Survey (CLDS) dataset from 2018 and relied on a regression model to examine the effects of the built environment, work environment, and subjective perception on the mental health of Chinese citizens. It also considers the circumstances of both migrants and local residents.

**Results:**

This study revealed significant correlations between mental health and greening space rate, road network density, commuting time, work feelings, community trust, economic satisfaction, and other factors. Additionally, the mental health of local residents was shown to be significantly affected by community security, while it shows no significance in migrants. Furthermore, a significant spatial autocorrelation was found in terms of mental health within the central and eastern regions of China.

**Discussion:**

The findings of this study offer valuable insights that can be used to facilitate measures aimed at improving the mental health of residents and promoting the development of healthy cities.

## 1 Introduction

In recent years, urbanization in developing countries has rapidly accelerated owing to the significant growth of the global economy. Urbanization has a positive impact on residents’ quality of life by enhancing the quality of medical services and infrastructure available to them. However, the ongoing influx of people into urban environments means that it is estimated that by 2050, most of the global population will reside in cities; indicating citizens may suffer more pressures resulting in some potential mental problems, with the increasing living costs and employment competition, as well as the less opportunities of visiting green spaces and accompanying families. According to the World Health Organization (WHO), more than 700,000 people die due to suicide worldwide, and mental health problems are one of the major contributors (World Health Organization [WHO], 2021). Meanwhile, some research has also shown that approximately 792 million individuals worldwide experience mental illness, accounting for approximately 10.7% of the global population (Ritchie and Roser, 2023). On one hand, the continuous growing of mental problem results in a series of bad influences on medical system, including the rising loads of mental treatment services (Lozano et al., 2012). On the other hand, it is reported that mental health issues result in a minimum annual cost of US$1 trillion to the global economy (World Health Organization, 2019). The main goal of the 2030 Agenda for Sustainable Development (SDGs) is to “Ensure healthy lives and promote well-being for all at all ages,” including reducing one-third premature mortality from non-communicable diseases through prevention and treatment and promoting mental health and well-being (Transforming our World: The 2030 Agenda for Sustainable Development|Department of Economic and Social Affairs, n.d.). Thus, in light of the significant social and economic implications, it is crucial to thoroughly investigate the factors that affect the mental health of urban residents.

Previous research has extensively examined the impact mechanisms affecting mental health across various multidisciplinary fields, including sociology, psychology and geography. This has included investigating the driving factors, methods, and differences associated with mental health. First, studies on driving forces have mainly focused on environments (e.g., social, built, and work environments) (Lin and Yang, 2015; Silva et al., 2016; Shen et al., 2021) and perceptions (e.g., subjective environmental perception and satisfaction) (Mehrabian and Russell, 1974; Liu et al., 2019a). Similarly, certain researchers have endeavored to investigate different methods for measuring mental health and implementing new technologies (Cuijpers et al., 2009; Liu et al., 2021). Furthermore, research interest has been drawn to the examination of mental health disparities, encompassing distinctions between urban and rural areas and sex-based disparities (Shen et al., 2021). Although existing research has drawn some important conclusions regarding the impact mechanism of mental health, significant gaps remain. Prior research of mental health has mainly focused on specific groups, such as the elderly or the migrants, and has limited exploration of the feature of geographical spatial distribution. Thus, this study examines the spatial correlation and influencing mechanisms affecting residents’ mental health in different regions of China, based on large-scale population data obtained from the China Labor-force Dynamic Survey (CLDS). A variety of influencing factors of objective environment and subjective perception were employed, including the built environment, work environment, and economic satisfaction, etc. Furthermore, the analysis also considered the comprehensive effects of random groups, as well as the varying effects on migrants and local residents. The findings of this study will serve as a valuable resource for urban planners and decision makers, contributing to the enhancement of mental health among urban residents and helping to enable the further progress of cities in China.

## 2 Literature review

### 2.1 Urbanization, transition and mental health

Urbanization has made cities becoming the ultimate destination for population agglomeration (Vearey et al., 2020). As a result, the labor force characteristics of China’s population are undergoing gradual changes. Urbanization and demographic transitions often lead to a range of health issues. Some studies have suggested that individuals in good health are typically the primary participants in this transition (Chen, 2011). Nevertheless, health risks do not manifest immediately but rather accumulate over time due to low adaptability or ongoing exposure to high-risk environments. Migrant workers often tend to find themselves in low-paying, dirty, harsh, and dangerous jobs compared to local permanent residents. This disparity can be attributed to differences in educational levels and household registration systems (Yue et al., 2013; Shen, 2017). In contrast to physical health issues, mental health concerns are often less apparent and more challenging to detect. Mental health disorders can generate various challenges and risks, including financial difficulties (Anand et al., 2018; Richardson et al., 2021), lethargy (Fibbins et al., 2018), depression, and shorter lifespans (Stubbs and Rosenbaum, 2018). Thus, exploring the mechanisms that influence mental health has important practical implications. Sweetser first introduced the concept of mental health (Sweetser, 1850). This definition has since been further developed through ongoing research (Gamm et al., 2010). As per the definition put forward by the World Health Organization in 2019, mental health in this study is defined as a state of well-being in which an individual can realize their own potential, cope with the normal stresses of life, work productively, and contribute to the community.

### 2.2 Measurement of mental health

As an abstract concept, most researchers generally choose to express mental health using multiple indicators. Consequently, psychological scale tools have emerged as the primary method for diagnosing mental health conditions. Internationally recognized scales that are frequently used include the Symptom Checklist-90 (SCL-90), the Positive and Negative Affect Schedule (PANAS) and the Self-Rating Depression Scale (SDS), etc. On the above mental health scales, the reliability and validity of SCL-90 which is recognized as a classical scale have been tested in China (Zhong et al., 2013), while SDS is difficult to evaluate depression with severe retardation symptoms, and the evaluation effect is poor for people with low educational level or slightly poor intellectual level (Guan et al., 2022). Meanwhile, PANAS has the limitations of an ordinal scale such as low precision and unsuitability for using with parametric statistics (Medvedev et al., 2023). In addition, with the development of data acquisition technology and hardware matching, the information collection mode of mental health has expanded to include wearable electronic devices with sensors (e.g., watch, helmet) and other devices to monitor ecological instantaneous assessments, physiological functions, environmental parameters, physical behavior (Fahrenberg et al., 2007), ultimately forming a longitudinal dataset that allows for the real-time collection, but it needs a long cycle of time consumption. Finally, after comparing the features of the previous scales, Depression Self-Assessment Scale (CES-D20) is employed in this paper to measure mental health, because of its high quality of operability, reliability and recognition degree (Bharadwaj et al., 2017).

### 2.3 Determinants of mental health

The determinants of mental health in previous researches have been mainly summarized into three aspects: built environment (e.g., land development intensity, greening space rate, road network density), work environment (e.g., commuting time, employment status), and subjective perception (e.g., community trust, economic satisfaction, and community security). The influence of these factors is usually related to the unequal distribution of social resources (Marmot et al., 2013). Especially the living conditions of the public, including built environment by which residents are surrounded and the working environment where activities are carried out may make people prone to exert different psychological feelings, thereby affecting people’s mental health (Braveman et al., 2011). Therefore, it is important to study the influencing mechanism of built environment, work environment and subjective perception on mental health.

On the one hand, the built environment, as an activity platform for residents’ daily lives, not only directly affects physical activity and active transportation (Smith et al., 2017), but is also indirectly related to the potential mental health risks caused by unreasonable housing and infrastructure. This has been confirmed in several studies (Melis et al., 2015; Kim and Yoo, 2019; Stone et al., 2022). At the same time, green space can provide important health and environmental services, reduce economic and social inequalities, reduce the mental stress of urban residents, and provide people with better well-being and mental health (Nutsford et al., 2013; Barton and Rogerson, 2017). On the other hand, the impact of the work environment has also gradually come to receive greater attention with regard to its impact on mental health. Poor working conditions (e.g., working overtime, basic salary) can generate stress, fatigue and other negative emotions (Novaco et al., 1979; Evans, 2003; Gatersleben and Uzzell, 2007); while long-term exposure to dangerous workplaces (e.g., to air and noise pollution) can lead to increased health risks (Lundberg, 1976; Feng and Boyle, 2014; Böcker et al., 2016). Long working or commuting hours also reduce the time available for exercise, relaxation, and communication with family and friends, and increase feelings of isolation (Lyons and Chatterjee, 2008; Christian, 2012; Mattisson et al., 2015). Therefore, the improvement of environmental factors is crucial not only to promoting healthy physical activity and reducing the risk of chronic diseases such as obesity and cardiovascular disease, but also to providing people with the opportunity for psychological recovery and spiritual release, thus promoting better mental health and improving overall happiness (Zhao et al., 2022).

Furthermore, subjective perceptions are also important to mental health. It is believed that subjective environmental perceptions affect people’s emotions and behavioral choices (Wang et al., 2019; Liu et al., 2019a). If an environment can promote positive perceptions, people will be more inclined to continue engaging in activities in that environment (Mehrabian and Russell, 1974). Previous research has shown a correlation between subjective environmental perception and healthy activities (e.g., physical activities and social interactions) (Astell-Burt et al., 2014; Cohen-Cline et al., 2015). However, it is worth noting that subjective environmental perception is usually based on the objective environmental conditions, which guide individuals to produce corresponding behaviors (Atari et al., 2009; Liao et al., 2015). Thus, subjective environmental perception may act as a mediator between the environment and behavior, a fact that has been further verified across a number of different theoretical frameworks (Kremers et al., 2006; Sallis et al., 2006). From previous studies, it can be seen that the influencing factors of mental health mostly lie in the relationship between environment and mental health or subjective perception and mental health, and rarely have the relationship between various aspects and mental health at the same time. Therefore, this paper starts from the three aspects of built environment, work environment and subjective perception, and discusses the relationship between these variables and mental health. I hope it can provide some help for future research.

## 3 Data and methodology

### 3.1 Study area and data sources

**Figure 1:**
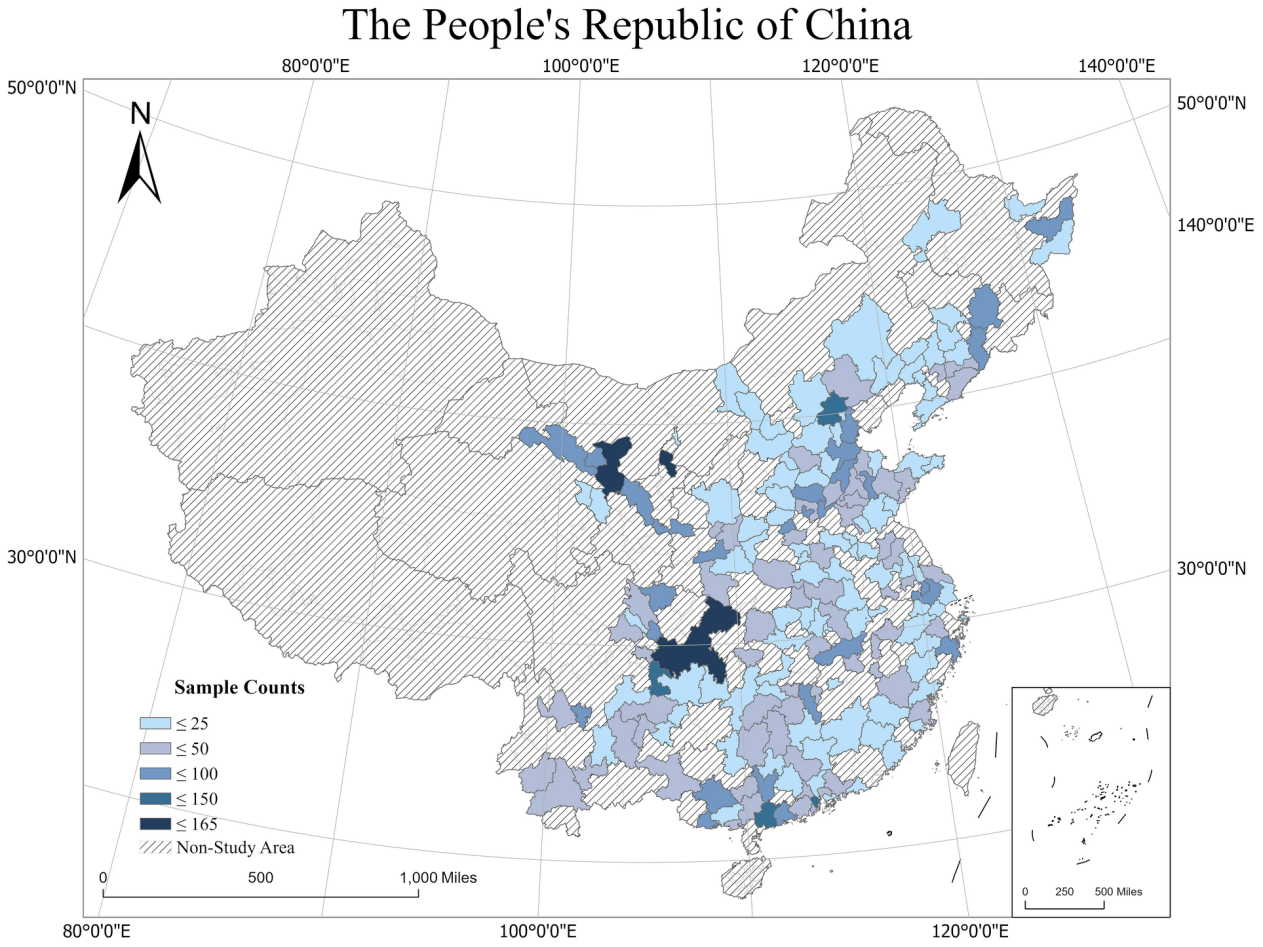
Study areas.

This study uses data from the 2018 CLDS (Liu et al., 2019b; Huang et al., 2023) conducted by the Centre for Social Science Investigation at Sun Yat-sen University. The CLDS project was the first interdisciplinary large-scale tracking social survey on labor status in China, focusing on the status quo and changes in China’s labor force. The project covers education, work, migration, health, social participation, economic activities, grassroots organizations, and other research topics. The project sample population covers 28 provinces and cities in China (with the exceptions of Hong Kong, Macao, Taiwan, Tibet, Hainan, and Xinjiang), and the survey object is the entire labor force (family members aged 15–64 years) within the sample households. This study used survey data from the CLDS in the 2018 specifically as the total sample. The investigation included individual self-assessments of mental health, lifestyle, work environment, and economic status. Based on the needs of the study, the study initially excluded samples with missing or invalid self-rated mental health scale, number of Hukou transfer, and other key indicators. Among the remaining samples, there were 623 missing values for the variable “fun to work” (9.63%) and 481 missing values for the variable “part-time job” (7.44%). Subsequently, multiple imputation was employed to address the missing values, resulting in a complete database with a sample size of 6,464. The sample size distributions in different regions are shown in Figure 1.

Table 1 shows the sociodemographic characteristics of the total sample and subsamples (migrants and local residents) used in this study. This study defines the local residents are those who have not relocated in the previous 6 months, while migrants are those who have left their current place of residence over 6 months. First, in the total sample, the mean value of age was 46.5, and the proportions of male and female respondents were 53.6 and 46.4%, respectively, of which 86.6% were married and 75% had zero Hukou transfers. Based on the socioeconomic characteristics of the research participants, the proportion of people satisfied with their employment status was relatively high (92.6%). The proportion of people who did not have part-time jobs was 92.7%. Among them, the proportion of respondents with higher education qualifications was generally low, at only 8.4%. In the subsample, the average age among the migrants and the local residents were 46.41 and 47.73. The proportion of female among the migrants was relatively high (72.8%), as was the proportion of male among local residents (60.4%). In terms of marital status, a high proportion of both the migrants and the local residents were married, 91.5 and 86.5%, respectively. Furthermore, both migrants and local residents reported a relatively high level of their employment status, at 91.8 and 92.8%, respectively.

**Table 1:** Sociodemographic characteristics of the total sample and subsamples.

**Variables**	**Total**	**Migrants**	**Local residents**
**Number of samples**	6,464	1,322	5,142
**Age (years)**			
Mean value	47.22	46.41	47.73
**Gender (%)**			
Male	53.6	27.2	60.4
Female	46.4	72.8	39.6
**Marital status (%)**			
Married	86.6	91.5	86.5
Unmarried	13.4	8.5	13.5
**Hukou status (frequency)**			
Mean value	0.25	1.18	0.01
**Employment status (%)**			
Employed	92.6	91.8	92.8
Unemployed	7.4	8.2	7.2
**Part-time job (%)**			
Yes	7.3	5.8	7.6
No	92.7	94.2	92.4
**Education level (%)**			
Low	69.7	62.3	71.6
Middle	21.9	23.3	21.6
High	8.4	14.4	6.8

### 3.2 Variables

Based on previous research, we selected three aspects that impact alterations in individuals’ mental health: built environment, work environment, and subjective perception. These dimensions were taken directly from the CLDS 2018 questionnaire and government-accessible data (National Bureau of Statistics Urban Socioeconomic Survey Department, 2019).

#### 3.2.1 Dependent variable

The dependent variable in this study was mental health. This study used the Depression Self-Assessment Scale (CES-D20) to measure mental health (Qiu et al., 2019). The Kaiser-Meyer-Olkin (KMO) value of the scale in this study was 0.971, with a significant Bartlett’s test of sphericity. Five of the 20 items on the mental health scale were daily behaviors that indicated poor mental states, and 15 of the items were frequent negative emotions that could have happened in the previous week. The scale uses a four-point scoring system 1 = Almost Always, 5–7 days per week; 2 = Often, 3–4 days per week; 3 = Rarely, 1–2 days per week; and 4 = Almost Never, less than one day per week; Higher scores on the overall scale, which range from 20 to 80, indicate that respondents’ mental health has improved over the previous week. Cronbach’s α of the mental health subscale was 0.947, indicating the reliability of the research measurement (Table 2).

**Table 2:** Measured variables of mental health.

**Constructed dimensions**	**Cronbach’s α**	**Cronbach’s α based on standardized terms**	**Variables**
Mental health	0.947	0.950	Worried about some small matters.
			Do not want to eat, have a bad appetite.
			Even with the help of family and friends, I still cannot get rid of the bitterness in my heart.
			I do not think it’s as good as most people.
			Unable to concentrate when doing things.
			Feeling down.
			Feeling that doing anything takes a lot of effort.
			Feeling hopeless about the future.
			Feeling like my life is a failure.
			Feeling scare.
			Poor sleep.
			Feeling unhappy.
			Speaking less than usual.
			Feeling lonely.
			Feeling that people are not very friendly to oneself.
			Feeling that life is meaningless.
			Crying.
			Feeling nervous.
			Feeling that people do not like themselves.
			Feeling that life cannot continue.

#### 3.2.2 Explanatory variable

This study divided the explanatory variables into three dimensions: built environment, work environment, and subjective perception. Built environment factors mainly include greening space rate, the number of hospitals, land development intensity, road network density and particulate matter 2.5 (PM2.5) index. Land development intensity refers to the proportion of total construction land in the administrative area. The work environment includes the variables of commuting time, employment hours (part-time vs. full-time), and employment status. Commuting time is expressed as the time it takes for a respondent to commute daily during their current or most recent job, while employment status is defined as either employed or unemployed. In addition, we divided participants’ subjective perceptions into work feelings, community trust, economic satisfaction, and community security. Work feelings refers to a 5 items scale related to work. The scale uses a five-point scoring system (1 = Not Satisfied; 2 = Not very Satisfied; 3 = Generally Satisfied; 4 = Relatively Satisfied; and 5 = Very Satisfied;) Community trust refers to a 9 items scale related to the trust to other different people. The scale uses a five-point scoring system (1 = Totally Unreliable.; 2 = Relatively Unreliable; 3 = Hovering Between reliable and unreliable; 4 = Relatively Reliable; 5 = Totally Reliable). Community security refers to the respondents reporting feeling in their community. The scale uses a four-point scoring system (1 = very unsafe; 2 = not very safe; 3 = relatively safe; and 4 = very safe).The values of Cronbach’s α of work feelings ( α =0.846) and community trust ( α =0.735) are presented in Table 3.

**Table 3:** Measured variables of work feelings and community trust.

**Constructed dimensions**	**Cronbach’s α**	**Cronbach’s α based on standardized terms**	**Variables**
Work feelings	0.846	0.849	Safety at work
			Working environment safe
			Working hours
			Fun to work
			Overall job satisfaction
Community trust	0.735	0.743	Family
			Relatives, Friends
			Neighbors
			Fellow countrymen
			Classmates
			Strangers
			Colleagues
			Businessmen, Customers
			Believers

#### 3.2.3 Control variables

This study employs age, gender (male or female), marital status (married or unmarried), Hukou transfer, and education level (low, middle or high) as control variables. Hukou transfer refers to the frequency of migration. Low education refers that the respondent’s educational attainment was junior high school and below. Middle education refers that the respondent’s educational attainment was between senior high school and college. High education refers that the respondent’s educational attainment was bachelor’s degree and above. The scientific authenticity of this study was guaranteed by controlling the variables.

### 3.3 Research methods

Considering the possibility that mental health is affected by spatial differences in the environment and subjective perceptions (Zhao et al., 2022). In this study, a single-level linear regression model was used to measure the impact mechanism on mental health. This study selected mental health as the dependent variable, used the Ordinary Least Squares (OLS) (Choi and DiNitto, 2013; Mersky et al., 2013) method to establish a linear regression model, and measured the impact of the built environment, work environment, and subjective perception of mental health. The resulting expression is as follows:


(1) $$Y=\alpha_{0}+\overset{n}{\underset{i=1}{\sum }}\beta_{i}x_{i}+\varepsilon$$

In Eq. 1, where Y represents the dependent variable, that is, mental health, $x_{i}$ represents the independent variables (including the built environment, work environment, and subjective perception) and control variables (including age, gender, marital status, hukou transfer and education level), $\beta_{i}$ represents its correlation coefficient. And $\alpha_{0}$ represents intercept, ε is a normal distribution in which the error obeys a mean value of 0.

## 4 Results

### 4.1 Spatial distribution characteristics

**Figure 2:**
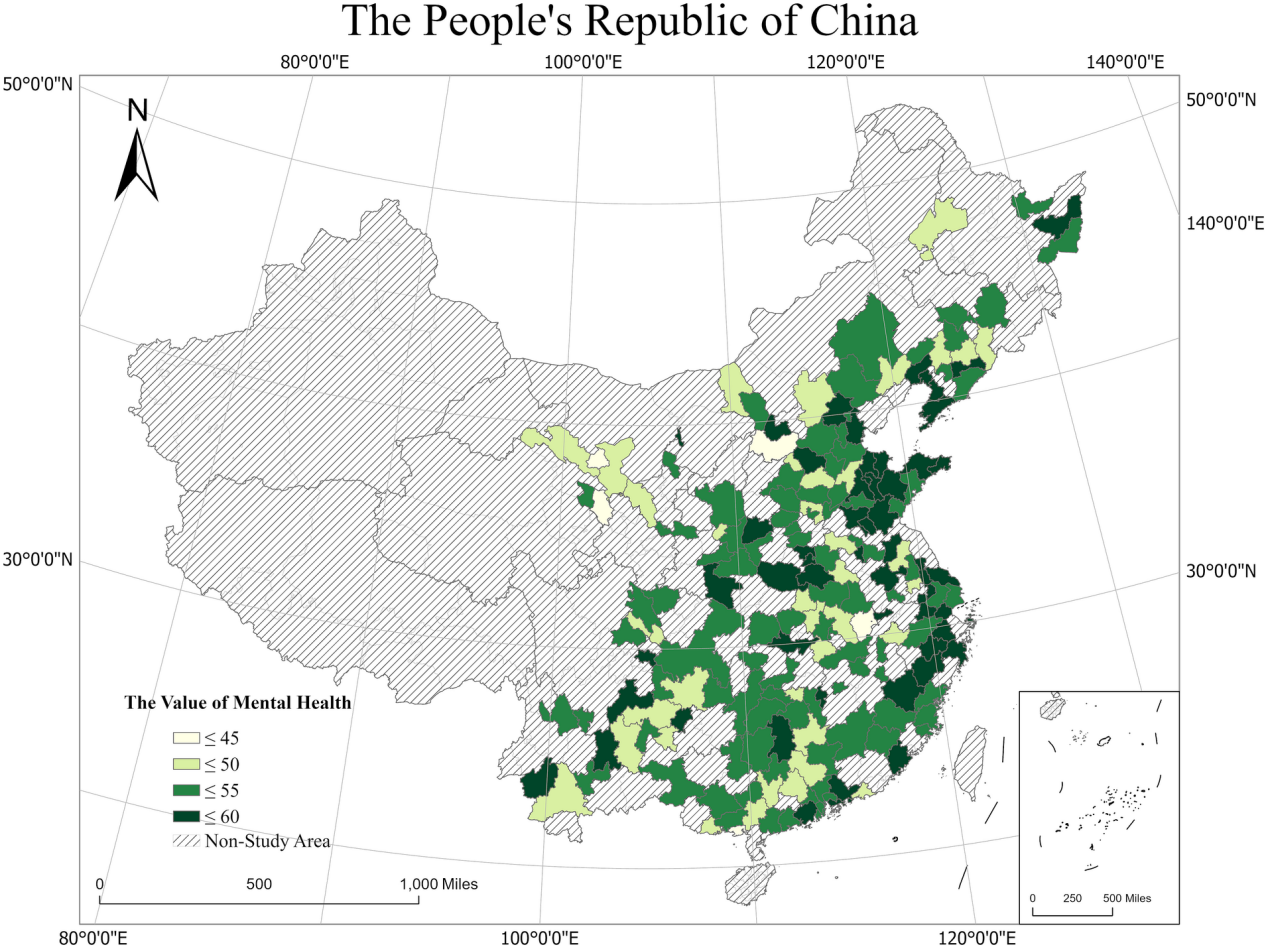
Spatial distribution of average mental health levels.

The average mental health scores are shown in Figure 2. Overall, the average mental health scores gradually increased from the western to the central and eastern regions. The average mental health scores of Jinzhou, Yingkou, Tongling and Taizhou were relatively high, indicating that the residents of these areas have better mental health status, they are happier, more optimistic and more resilient. While respondents from Beihai, Jinchang and Yangquan had relatively low mental health score, they may face more stress and psychological barriers in their daily lives.

**Figure 3:**
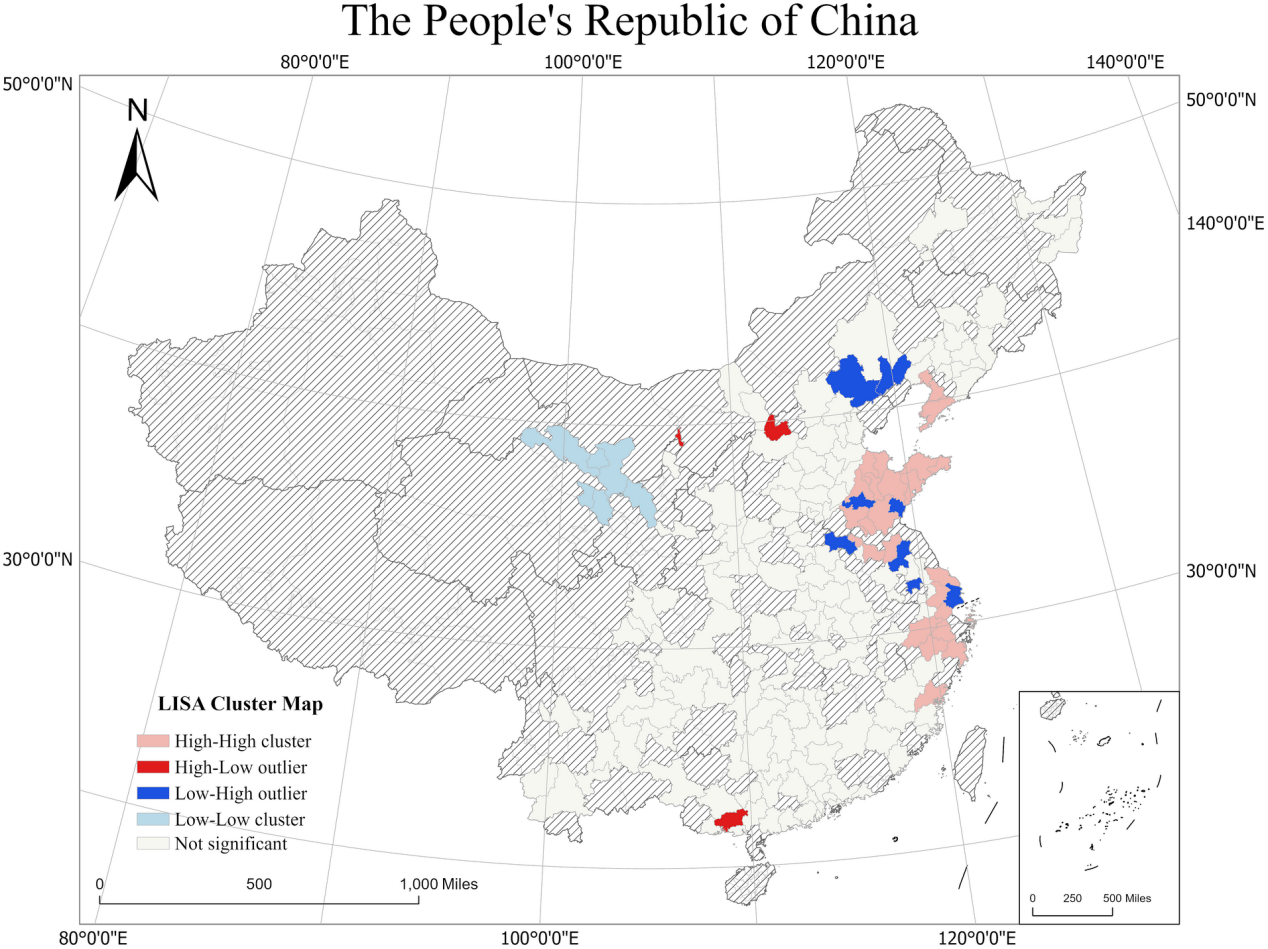
Spatial autocorrelation of the mental health.

To explore the spatial differentiation and autocorrelation characteristics of mental health in city scale, Global Moran’s *I* (Moran, 1950) and Local Moran’s *I* (Anselin, 1995) were implemented. The Moran ‘s I index was 0.103 with a *Z*-score of 4.162 and a value of *p* <0.01, indicating that the mental health in study area has a positive correlation. As shown in Figure 3, we further explored the spatial differentiation characteristics of mental health using Local Indicators of Spatial Association (LISA) clustering at the city scale in the study area. The results of the LISA clustering significance test showed that, first, eastern regions, for example, cities in Shandong province, Hangzhou, Suzhou, Nantong in Yangtze River Delta and Dalian, Yingkou in Liaodong Peninsula generally showed a positive local spatial correlation with mental health and belonged to high-high clustering areas. This indicates that the mental health scores of the residents in and surrounding cities were higher overall, and they may generally have a better mental health status. Second, cities such as Shanghai, Chengde, Zhengjiang and Huai’an are low-high clustering areas, indicating that people living in those areas may have worse mental health status than surrounding cities. The high-low clustering areas including Shuozhou and Qinzhou, indicating that the mental health scores in these areas were higher than those in the surrounding areas. Low-low aggregation areas mainly included in cities in Gansu and Qinghai provinces such as Wuwei, Zhangye and Xining, indicating that the residents in these areas have relatively poor mental health.

### 4.2 Results from baseline model

As shown in Table 4, regression analysis of the baseline model shows that there is a significant positive correlation in subjective perception factors, among which the coefficients of work feelings, community trust, economic satisfaction and community security are 0.235, 0.254, 1.525, and 0.921, respectively. This means that better subjective feelings contribute to better mental health status, because better work experiences, friendly interpersonal relationships, higher income and higher sense of security mean lower levels of work stress and greater enthusiasm for life, a finding which is consistent with those recorded in previous studies (Jakobsen et al., 2022; Shields-Zeeman and Smit, 2022). Second, in terms of the built environment, an increase in the greening space rate causes an increase of 0.001 point of mental health. Generally, a higher green space rate means higher environmental quality and more parks and other leisure places to promote social activities among residents, especially the elderly (Home et al., 2012; Bertram and Rehdanz, 2015). An increase of Road network density also causes an increase of 1.153 point of mental health. A higher road network density may reduce vehicle speed in the community, decreasing peoples’ concerns regarding traffic accidents. Higher road network density can also improve accessibility to the community, providing more walking options to make it easier to reach service facilities (Tao et al., 2020). In addition, in terms of the work environment, commuting time had a significant negative correlation (*p* < 0.001) with mental health. For control variables, age, marital status and low education have significant negative correlations with mental health with coefficients of 1.547, −0.055, and −0.830, respectively. This result indicates that mental health may decline when people get aged, get married or have a low education level.

**Table 4:** The results of regression for total sample.

	**Coeff.**	**S.E.**	***t*-value**
**Sociological attributes of population**			
Age	−1.547^***^	0.220	−7.04
Gender	0.136	0.156	0.88
Marital status	−0.055^***^	0.010	−5.74
Hukou transfer	−0.289	0.202	−1.43
Low education	−0.830^***^	0.308	−2.70
Middle education	0.000	0.000	0.00
High education	−0.077	0.402	−0.19
**Built environment**			
Greening space rate	0.044^***^	0.013	3.47
Number of hospitals	0.003	0.011	0.29
Land development intensity	−0.005	0.016	−0.28
Road network density	1.153^***^	0.191	6.03
PM2.5 index	0.015	0.011	1.34
**Work environment**			
Commuting time	−0.011^***^	0.003	−3.14
Part-time job	−0.377	0.413	−0.91
Employment status	0.569	0.408	1.40
**Subjective perception**			
Work feelings	0.235^***^	0.034	6.90
Community trust	0.254^***^	0.035	7.28
Economic satisfaction	1.525^***^	0.113	13.51
Community security	0.921^***^	0.178	5.19
_cons	38.583^***^	1.522	25.36
*R* ^2^	0.104		
*N*	6,464		

*p < 0.10; **p < 0.05; ***p < 0.01.

The mean of the mental health scores for local residents and migrants are 52.52 and 51.58, respectively. The *t*-test results for the differences between local residents and migrants’ groups are statistically significant, as the significance (Sig.) of Hukou transfer is higher than 0.05 for Levene’s test and less than 0.001 in the *t*-test (Table 5). The mental health of local residents’ group (*t* = 3.417) seems higher than those of migrants group (*t* = 3.356). Thus, the following section further delves into the impact mechanism of mental health and explores the impact of the built environment, work environment and subjective perception on mental health between migrants and local residents.

**Table 5:** Independent sample t-test results for mental health.

**Dependent variable: mental health**				**Levene’s test**			***t*-test**			
**Demographic**		**Mean**	**S.E.**	**Mean of S.E.**	***F***	**Sig.**	***t***	**Sig.** **(Two Tail)**	**Mean**	**S.E.**
**Gender**	Female	53.09	8.424	0.143	39.976	0.001	7.387	0.001	1.642	0.222
	Male	51.45	9.451	0.173			7.326	0.001	1.642	0.224
**Marital status**	Unmarried	51.83	9.696	0.342	10.985	0.001	−1.692	0.091	−0.571	0.337
	Married	52.40	8.840	0.118			−1.579	0.115	−0.571	0.361
**Employment**	Employed	52.33	9.009	0.116	0.571	0.450	0.099	0.921	0.042	0.426
	Unemployed	52.29	8.212	0.376			0.108	0.914	0.042	0.394
**Education**	Lower education	52.02	9.068	0.122	33.998	0.001	−6.526	0.001	−2.021	0.310
	Higher education	54.05	8.063	0.258			−7.081	0.001	−2.021	0.285
Hukou transfer***	Local residents	52.52	8.888	0.124	1.805	0.179	3.417	0.001	0.942	0.276
	Migrants	51.58	9.163	0.252			3.356	0.001	0.942	0.281

*, **, and *** Represent significance levels of 5, 1, and 0.1%, respectively.

### 4.3 Migrants vs. local residents

As shown in Table 6, by constructing a subsample model for migrants and local residents groups, we found that work feeling (*p* < 0.01), community trust (*p* < 0.01) and economic satisfaction were all significantly and positively correlated with mental health. However, there are some differences in the factors that affect the mental health of migrants and local residents. For the migrants group, the coefficient of economic satisfaction ( β = 2.034, *p* < 0.001) is higher than the local residents group ( β = 1.393, *p* < 0.001). The reason for this phenomenon may be that migrants usually desire a higher level of income to settle down, so they face more pressure, creating a significant psychological burden (Zhao, 1999), especially for female migrants (Bardasi and Francesconi, 2004; Jackson et al., 2019). Better economic satisfaction may help migrants improve their mental health. Besides, among local residents, community security was found to be significantly related to mental health ( β = 1.095, *p* < 0.001), while the migrants group shows no significance among community security. Furthermore, for the local residents group, the coefficient of community trust ( β = 0.270, *p* < 0.001) is higher than migrants group ( β = 0.185, *p* < 0.01). The scope of activities among local resident communities is relatively stable, meaning where they live is a very important consideration. The safety and trust of the community are important requirements for local residents who usually have children, and children are usually more susceptible to potential artificial hazards (e.g., violence) (Jackson et al., 2019) or natural hazards (e.g., pollution) (Tortorella et al., 2022). For the built environment and work environment, both the migrants group and the local residents group show positive significance in the greening space rate and road network density to mental health. Previous studies found that community green spaces can alleviate the homesickness of the migrants by increasing social cohesion and have a positive effect on the mental health, and local residents may pay attention to the greening of their living environment due to their long-term stable living characteristics (South et al., 2018; Yang et al., 2020; Zhang et al., 2022). Commuting time has a negative impact on mental health. Commuting drains energy and attention. Long-time commutes to work are likely to be much more tiring and stressful than shorter time commutes (Evans et al., 2002). Excessively long commutes may reduce people’s mental health and may increase the risk of depression (Wang and Liu, 2022). For other variables, the number of hospitals, land development intensity, PM2.5 index, part-time or not and employment status show no significance.

**Table 6:** The results of regression for subsamples.

	**Migrants**			**Local residents**		
	**Coeff.**	**S.E.**	***t*-value**	**Coeff.**	**S.E.**	***t*-value**
**Sociological attributes of population**						
Age	−1.608^***^	0.590	−2.72	−1.433***	0.246	−5.83
Gender	−0.067	0.374	−0.18	0.165	0.171	0.96
Marital status	−0.052**	0.024	−2.11	−0.057***	0.010	−5.41
Hukou transfer	−0.105	0.463	−0.23	0.599	0.887	0.67
Low education	−0.732	0.741	−0.99	−0.621	0.401	−1.55
Middle education	−0.000	0.000	−0.00	0.173	0.462	0.38
High education	−0.045	0.844	−0.05	−0.000	0.000	−0.000
**Built environment**						
Greening space rate	0.054**	0.026	2.10	0.040***	0.015	2.71
The number of hospitals	0.004	0.020	0.20	0.002	0.013	0.17
Land development intensity	−0.030	0.035	−0.85	0.007	0.019	0.37
Road network density	1.000***	0.383	2.16	1.270***	0.223	5.68
PM2.5 index	−0.009	0.026	−0.34	0.017	0.012	1.42
**Work environment**						
Commuting time	−0.016**	0.007	−2.21	−0.009**	0.004	−2.30
Part-time or not	1.139	1.031	1.11	−0.693	0.451	−1.54
Employment status	1.459	0.887	1.65	0.261	0.461	0.57
**Subjective perception**						
Work feelings	0.230***	0.078	2.95	0.239***	0.038	6.32
Community trust	0.185**	0.079	2.35	0.270***	0.039	6.94
Economic satisfaction	2.034***	0.251	8.09	1.393***	0.127	11.00
Community security	0.154	0.417	0.37	1.095***	0.197	5.56
_cons	37.428^***^	3.494	9.48	38.644^***^	1.714	22.55
*R* ^2^	0.117			0.103		
*N*	1,322			5,142		

*p < 0.10; **p < 0.05; ***p < 0.01.

## 5 Conclusion and discussions

Using the 2018 CLDS database, this study explored the impact mechanisms between the built and work environment, subjective perception, and mental health by constructing a regression model and exploring the differences between migrants and local residents while exploring the spatial correlates of mental health. The findings of this study are as follows. On the one hand, the increase of greening space rate, road network density, rework feelings, community trust, and economic satisfaction will benefit the mental health of both migrants and residents, while the increase of commuting time will be harmful to mental health. On the other hand, there are also some differences in the impact of different factors on the subsamples of migrants and local residents, respectively. Firstly, the greening space rate and community trust have a significant impact on residents’ mental health, especially for local residents. Meanwhile, it is noteworthy that community security is found to be significantly correlated with the mental health of local residents, and shows no significance in migrants. In addition, individuals’ mental health showed obvious spatial differences, with the areas with larger differences mainly located in central-eastern China.

Based on these results, the following measures are recommended to improve mental health status in China: First, individuals’ work experience and economic income must be improved. One means of achieving this is by gradually increasing the proportion of labor remuneration in primary distribution through national policy support, mobilizing the enthusiasm of workers across the whole of Chinese society, and further promoting workers’ level of income in the process (Li et al., 2022). However, enterprises also need to create a good work environment for employees, regularly conduct mental health examination activities, provide reasonable social security, and promote the mental health of workers(De Moortel et al., 2018). Second, infrastructure must be improved and the volume of community exchanges must be increased. Urban planning managers should conduct reasonable community planning and enrich public service facilities to provide residents with more space for interaction and to enhance their sense of security in the community (Liu et al., 2019a). Furthermore, green areas in urban public spaces should be increased to improve urban construction and management, especially the public health environment. Governments at all levels should pay more attention to healthy cities and increase support for health services, while increasing investment in community health service institutions, standardizing the management of public health institutions, and optimizing the construction of mental health workforces. In addition, community staff also need to strengthen the publicity of health service policies, popularize healthy lifestyles to the public, improve residents’ mental health awareness, and at the same time to meet the residents’ mental health needs, mobilize their motivation and participate in the community health service work. This improves the natural environment, social environment and health services in cities, creating better and healthier cities (Maas, 2006).

This study has a number of limitations. First, the data used were cross-sectional data from the year 2018, and the exploration of time evolution was limited. In future, spatiotemporal differences could be explored using CLDS data from different years. Second, this study used regression to explore the impact mechanisms affecting mental health status among Chinese residents; in the future, attempts can be made to assess these same variables from multiple perspectives using a variety of different models, such as the mediation model. Moreover, based on the data obtained, this study divides the independent variables into three dimensions: work environment, built environment, and subjective perception. Thus, the possibility of a different impact mechanism that affects mental health status can be explored through a more enriched dataset.

## Data availability statement

The original contributions presented in the study are included in the article/supplementary material, further inquiries can be directed to the corresponding author.

## Ethic statement

Ethical review and approval was not required for the study on human participants in accordance with the local legislation and institutional requirements. Written informed consent from the patients/participants or patients/participants legal guardian/next of kin was not required to participate in this study in accordance with the national legislation and the institutional requirements.

## Author contributions

ZF: Conceptualization, Methodology, Writing – original draft. YL: Methodology, Software, Writing – original draft. CC: Formal analysis, Validation, Writing – original draft. JJ: Resources, Writing – original draft. LD: Investigation, Resources, Writing – original draft.
